# “The Perception of Visiting Holocaust Sites on Undergraduate Students Learning Process”

**DOI:** 10.1007/s10755-022-09606-9

**Published:** 2022-05-13

**Authors:** Anna Bussu, Peter Leadbetter, Michael Richards

**Affiliations:** 1 Department of Law & Criminology, Edge Hill University, Ormskirk, UK; 2 Faculty of Health, Social Care and Medicine, Edge Hill University, Ormskirk, UK

**Keywords:** Qualitative research, Student learning process, Higher education

## Abstract

This paper presents the main findings of a qualitative research project. The aim of the research was to explore undergraduate students’ perceived knowledge acquisition and awareness of the Holocaust, after visiting Auschwitz concentration camp in Poland. The qualitative study (focus groups & semi-structured questionnaires) involved three cohorts of students and lecturers from a university in the North West of England. The participants visited the Holocaust-related sites in Poland from 2016 to 2019. Findings indicate that students’ who actively engaged in visits to Holocaust related sites developed knowledge and awareness of the Holocaust. For many participants, this knowledge and awareness was facilitated via a reflective process that enabled empathic connection between these historical events and the students. The study also highlighted practical strategies that could be implemented to enhance the experience for future cohorts of undergraduate students visiting holocaust related sites. By adding to the limited literature on Holocaust education with undergraduate students, the study highlighted the importance and directions for future research in this area to inform future pedagogic practice.

## Introduction

The Holocaust is one of the foremost historical and tragic events of the 20th Century. Although atrocities between 1939 and 1945 were committed against a number of political, religious and ethnic groups including Roma people, homosexuals, and people with physical and intellectual disabilities in the name of national socialism, the Holocaust in this context refers to the Nazi-German genocide of approximately 6 million European Jews (Bartov, 2015; Reis et al., 2019).

This led Western scholars and policy makers (since 1945) to become increasingly sensitive to the need to educate society about the dangers of exclusionary institutional structures and genocidal social policies. For example, in response to this, state systems of education prepared curricula and pedagogies, which, in addition to teaching historical facts about the Holocaust, have focused on moral awareness and enhancing the capacity for social criticism (Cowan & Maitles, 2011; Gallant & Hartman, 2001).

In Europe since the 1990s, the Holocaust has been commemorated by creating symbolic and pedagogical places such as the Terence Warsaw Ghetto Museum. This museum has an Educational Training Centre that focuses on knowledge exchange as well as the moral/ethical issues related to the Holocaust. Other sites include the Jewish History Museum of Prague, which houses the "Lost Neighbours" project. This project aims to encourage students to examine the long history of Jewish communities in Europe (Davis & Rubinstein-Avila, 2013). In addition, the 1990s saw the establishment of the intergovernmental body, the International Holocaust Remembrance Alliance (IHRA), with the aim to bring together the support of political and social leaders to promote the need for Holocaust education and remembrance. The IHRA currently has 31 member countries and is an alliance where members are expected to implement policies that support Holocaust education and remembrance in their respective countries, but also share good and best practices in promoting Holocaust education. Within this organisation there are committees that specialise in specific issues relating to the Holocaust such as Holocaust denial and the more recent Roma genocide. To increase knowledge and awareness of the Holocaust the organisation provides access to archives, educational resources, research and memorial days.

Furthermore, since 1988, the International March of the Living (MOTL) (2021) has run an annual educational program, bringing individuals (adults/young adults) from around the world to Poland and Israel to study the history of the Holocaust and to examine the roots of prejudice, intolerance and hatred. The MOTL goal “is not so much to learn from or about history – but to enter into history” (cit in Alba, 2015, 126). Supporting an emotional experience during such programs is part of the learning expectations, offering to the participants some pedagogic approaches to encourage historical, critical, and ethical reflection in the present. However, there is little evidence to support and provide credibility to such claims.

The ‘Stockholm Declaration’ was the founding document that still affirms the guiding principles of the IHRA. Such principals include a focus on the Holocaust as changing civilisation, the relevance of the Holocaust in contemporary society, an explicit commitment to commemorate the victims, and education of the public supported by research (Kaiser & Storeide, 2018). The Stockholm Declaration was reaffirmed in 2020 to mark the 75th anniversary of the end of World War Two, in the context of fewer survivors (IHRA 2020 Ministerial Declaration, 2020). However, despite the work this organisation promotes, and the aims to promote Holocaust education, the past couples of decades have also seen the rise of ‘new anti-Semitism’ (Romeyn, 2020). In the UK, the Community Security Trust found increased anti-Semitic incidents in the UK in 2019, and the Home Office has found that Jewish people were the second most commonly targeted group for religious hate crime. Similar reports have been found in other European nations such as Germany, France and the USA. This would suggest that despite the efforts by European governments to educate the public about the Holocaust, such as promoting Holocaust Memorial Day and the development of education such as the Holocaust Memorial Day (HMD) Education pack in the UK (Davis & Rubinstein-Avila, 2013), anti-Semitism is a still a prominent part of society.

In the context of the UK and the education system, there are isolated cases of curriculum that address the Holocaust, however there is limited evidence in higher education of the impact of students visiting Holocaust sites. Projects that have been implemented within the UK, such as “The Holocaust Education Trust” in England, and the “Lessons from Auschwitz (LFA)” in Scotland, focused on encouraging students to organise Holocaust related activities in their schools and communities. Although research into the impact of the project has demonstrated development of student awareness in relation to racism, and human rights, few projects have encouraged student visits (or actively researched the impact) of Holocaust related sites (Cowan & Maitles, 2011).

### Issues with Holocaust Education

Educators and academics are faced with the challenge of how best to develop educational practices and projects to stimulate knowledge development, remembrance, moral and emotional engagement of the Holocaust (Kranz, 2014; Szejnmann et al., 2018). Studies have focused on the content of educational programs, ethical issues associated with teaching the Holocaust, or strategies to develop cultural memory and consolidate knowledge of this period (De Bruijn, 2018).

Previous research on Holocaust education has emphasized the relevance of learning processes during excursions to Holocaust memorial places, especially the affective/emotional impact on students experiential learning (Romi & Lev, 2007), their modes of understanding the Holocaust (Lazar et al., 2004a), and their Jewish identity (Lazar et al., 2004b). However, limited research has explored experiential learning related to Holocaust memorial places with tertiary (Higher Education) students. Furthermore, according to Cooke and Frieze (2015) the discussion of Holocaust fieldwork at tertiary level has often been descriptive and under-theorized (see Friedman, 2002). Hence, the educational benefit of visits to genocide related sites is inconclusive (Andrews, 2010), with research predominately focusing on the educational impact of such visits on school aged students or medical students (González-López & Ríos-Cortés, 2016; Reis et al., 2019).

The effective impact of excursion of Holocaust memorial places on students “learning” is still controversial. Ben-Peretz and Shachar (2012) highlighted a positive impact of experiential learning in Holocaust Education. Romi and Lev (2007) found positive impact on “Journey to Poland” highlighting that students (adolescents and young adults) active participation in an emotional-cognitive experience enables an “authentic acquaintance” with the Holocaust (Richardson, 2021).

It is from this social/emotional perspective that Holocaust education can develop the empathy of students (Nowell & Poindexter, 2018). That is, empathy of students can be developed by exploring historical events to facilitate ‘the process of cognitive and affective engagement with historical figures to better understand how people from the past thought, felt, made decisions, acted, and faced consequences’ (Endacott, 2014, p. 4). This connection to history, cognition and the affective is defined as historical empathy (Endacott, 2014).

In this context there is a limited qualitative research focus on undergraduate students learning processes and historical empathy development, connected with visiting Holocaust sites. This is supported by evidence from the United States and the UK, where a growing concern has emerged outlining the need to know the nature and impact of this experience on university students’ learning and/or development (Gallant & Hartman, 2001). Hence, research is required on the learning processes and pedagogic and social/emotional impact of visits to Holocaust related sites for university students.

### Historical Empathy Development in Higher Education

According to Endacott (2019; Endacott & Brooks, 2013) ‘Historical empathy’, grounded in a psychological conception, is a dual construct that includes cognitive and affective dimensions.

Endacott and Sturtz (2015) argued that the cognitive and affective elements need to be synthesised to provide both a practical and theoretical model. This process involves contextualising the actions of historical figures (& victims) by explaining and evaluating their actions in social and historical contexts (Savenije & de Briujn, 2017). That is, understanding the circumstances people faced, and how/why people made such decisions (Utami, 2019). This aligns to research that has explored “historical consciousness”, defined as “the understanding of the temporality of historical experience or how past, present and future are thought to be connected” (Glencross, 2015, 1), and his importance for students to Holocaust education (Porat, 2004).

The aim is to assist students in recognising and contextualising past perspectives by aligning curricular objectives to relatable student experiences in everyday life (Brooks, 2011; Endacott, 2010). Thus, historical empathy ‘needs to discern the difference between life in the present and life in a distant past while maintaining the possibility that past perspectives hold some validity’ today (Barton & Levstik, 2004 cited in Endacott & Brooks, 2013, 42). Thus, historical empathy can be limited as modern ways of thinking may not readily explain behaviour from the past and hence may be incompatible with modern perspectives (Endacott, 2014; Endacott & Sturtz, 2015).

In the context of Holocaust education (& this study), historical empathy aligns to Endacott and Brooks (2013, 43) conceptual framework. This includes:

- *historical contextualisation:* deep understanding of the period/phenomenon under investigation, including socio-political and cultural norms and the events that are happening concurrently
- *perspective taking:* understanding of another’s prior lived experience, principles, attitudes and belief in order how that people might have thought about the situation.
- *affective connection:* considerations for how historical figures’ lived experiences, situations or actions may have been influenced by their affective response.

To foster *historical contextualisation, perspective taking* and *affective connections* consistent with the concept, it has been argued that reflective teaching and practice is essential, and that lecturers need to assist students in making these cognitive and affective connections (Perrotta, 2018; Utami, 2019). Under such circumstances it has been argued that historical empathy can help students understand varied perspectives and tolerance of others via active learning processes and student engagement with historical events (see Barton & Levstik, 2004; Brooks, 2008; Endacott, 2010; Grant, 2001). That is, historical empathy focuses on making connections to the past and the understanding of historical circumstances and consequences (Endacott, 2014; Lee & Ashby, 2001). The authors of this paper adopt this conceptual approach and do not aim to refocus attention on the historical roots of empathy consistent with more philosophical approaches (see in this regard Retz, 2015, Collingwood & Gadamer cit in Retz, 2015).

### The Pedagogic Impact of Holocaust Education and Visits on Undergraduate Students’ Professional Identity

The current literature focuses more on the pedagogical impact of visits to Holocaust sites by secondary school children, with a lack of evidence examining the Holocaust and visiting Holocaust-related sites in higher education. This is surprising given evidence that the development of the historical empathy process can facilitate students’ active understanding and contextualisation beyond the classroom by fostering personal connections to historical events (Barton & Levstik, 2004; Endacott & Brooks, 2013).

Some research in higher education has focused on the link between students professional identity formation and Holocaust education (Adams et al., 2006; Ben-Sefer, 2006; González-López & Ríos-Cortés, 2016; Reppers & Breeze, 2007; Reis et al., 2019). Research indicates that visiting Holocaust sites assisted in developing medical trainees’ ethical understanding of issues such as prejudice, assisted reproduction, resource allocation, informed consent (González-López & Ríos-Cortés, 2016; Reis et al., 2019). This enabled exploration of medical trainees implicit bias and prejudices that shape relationships.

Central to the development of a student’s professional identity is a guided, emotional learning process, where lecturers guide the process of student self-reflection and ‘self-to-other connection’ (Endacott, 2014, 29). Despite this, there is a lack of evidence highlighting the link between the reflective processes of undergraduate students and Nazi and/or Holocaust education outside of medicine and nursing.

In this regard, the aim of this study was to explore the perceptions of visiting Holocaust sites on undergraduate student’s perceptions of learning processes. The study explored the development of historical empathy and reflection to support this process before exploring the factors (& research evidence) that could be implemented to improve the students’ pedagogical experience.

## Research Methodology

### Research Context and Participants

The researcher’s positionality included reflecting on their own philosophy, biases and assumptions as impacting on the research (Creswell, 1998). Although none of the researchers identified as members of the groups targeted for genocide (e.g., Jewish, LGBT, Gypsies) or aligned to the perpetrators (e.g., German) in the Holocaust, all researchers acknowledged that they have hidden assumptions and biases that are not neutral and cannot be completely separated from the research process. There is little doubt that all researcher’s experiences of working with vulnerable groups influenced the study by understanding the importance of providing a safe and supportive environment for open discussion.

The research was completed at a university in the North West of England. Three cohorts of students were involved in the pedagogic phase (see 2.3) and visited Holocaust related sites (*n*=44). The forty-four student participants included sixteen third year undergraduate students in the 2016/17 academic year, eighteen in the 2017/18 academic year and ten in the 2018/19 academic year. Of the forty-four students, thirty students (*n*=30) participated in the focus groups (twenty-six women and four men). Nine (*n*=9) lecturers were also involved in pedagogic phase delivery and visited Holocaust related sites. All lecturers completed semi-structured questionnaires. Three lecturers in the academic year 2016/17, three in the 2017/18 and three in the 2018/19 (*n*=9) (three women and six men).

The students’ undergraduate degree integrated the disciplines of psychology with the applied sociology of deviance to examine offending behaviour from the perspective of the victim and the perpetrator. The overarching aim of the degree is to facilitate a comprehensive understanding of offending, and the impact of offending on the individual and community. Throughout the programme the curriculum aims to facilitate the development of their professional identity and employment with crime-related service providers. Given the sensitive and complex nature of issues explored as part of the degree, emotional and interpersonal skill development is inherent in the program (Botvin & Griffin, 2004).

### Procedures

The project provided two phases:

1. Pedagogic content phase: lecture and classroom-based activity in preparation for the visit to Holocaust-related sites (see 2.4);
2. Qualitative research
3. Focus groups with student participants (post visit).
4. Semi-structured questionnaires with lecturers (post visit).

The pedagogic content phase was implemented according to Endacott and Brooks’ four phase for development of historical empathy. The aim is to foster *historical contextualisation, perspective taking* and *affective connections.*

- *Introductory phase* which aimed to introduce the historical situation and/or the historical figure(s).
- *Investigation phase* in which participants adopted primary and secondary sources to develop a deeper understanding of the historical context (reading books, watching historical movies, visiting Holocaust related sites).
- *Display phase* when, in this case, students actually visited Holocaust sites.
- *Reflection phase* to make connections between the past and the present. This phase invited students to consider how their personal views may have changed as a result of the experience.

The aim was therefore to not only engage in research, but to facilitate the process of historical empathy via reflection. This paper presents the findings from the qualitative research phase, however an understanding of the pedagogic content if firstly required.

### Pedagogic Content

#### Introductory and Investigation Phase

Students’ attended two classroom-based sessions prior to the visit. The sessions were aimed at fostering historical contextualization, perspective taking and affective connection to the Holocaust (Endacott & Brooks, 2013), via visiting and supported by a reflective process.

All lecturers who facilitated these sessions were experienced academics who understood the concept of historical empathy and had an in-depth knowledge of the Holocaust, and related context and socio-political factors. Classroom sessions therefore focused on the historical, political, socio-cultural and economic contextualization of factors that contributed to the Holocaust *(historical contextualization*). Sessions also focused on the historical context of Nazism, Nazi ideology and eugenics, the victims and life of Nazi concentration camps (specifically Auschwitz-Birkenau), stereotypes and prejudices on Jewish communities and antisemitism, human rights and victim’s perspectives/narratives (*perspective taking*).

In these sessions, students discussed their knowledge of, and the contemporary relevance of the lessons (& previous course learning) to the Holocaust. Open discussions and activities provided students with the opportunity to consider diverse opinions and were invited to reflect on the factors that influence a given perspective (Endacott, 2010). Such perspectives were supplemented by narratives from victims and perpetrators (*perspective taking*). This included the Oskar Schindler case study and later a visit to the Schindler’s Factory Museum. Students were also actively encouraged to watch “Schindler’s List” movie, directed/co-produced by Steven Spielberg and written by Steven Zaillian in 1993. The aim was to use the film to support students in developing empathic connections to historical events (Stoddard, 2008). According to Romi and Lev (2007) this preparation is essential in students emotionally before the journey and also for developing emotional ties with the subject.

#### Display and Reflection Phase

Soon after the pedagogic sessions took place (January 2017, January 2018, February 2019), students and lecturers visited Holocaust related sites in Poland for 4 days (*perspective taking* and *affective connections*). On the first day students visited the Jewish Ghetto (with tour guide) and the Schindler’s Factory Museum. The latter is a permanent exhibition which opened in 2010 in Krakow, Poland.

On the second day, and led by a tour guide, students and lecturers visited the “Memorial and Museum of Auschwitz-Birkenau”. Participants stopped at several sites and read testimonies from victims, complementary information about the site, or poems to commemorate the Holocaust victims. The aim was to support students learning processes by facilitating connections with sites as mediated spaces to allow young people to develop understanding, an emotional connection, and interpretations within their developing world view (Richardson, 2021).

The other two days included a travel day and one day “free-time” to explore the city of Krakow.

To supplement the reflective phase students were reminded of previous teaching and experiences of reflection on the course. That is, as 2^nd^ and 3^rd^ year students it was stressed to them that they are moving towards a process of becoming independent learners via critical reflection. This included revisiting the critical reflective process that includes questioning, clarifying, challenging, judging the credibility of sources, and solving problems by predicting probable outcomes (Lipman, 1988). To structurally support this process, students have a knowledge and practical understanding of the Gibbs reflective model (1988) and the associated phases of describing, assessing, analyzing and evaluating. Discussion also focused on the research process of the study, and hence using the student focus group as a formal way to reflect on the experience as a group.

In summary, the pedagogic aim of the experience was to foster self-reflection and knowledge on how the content applies to students’ own lives and values, and to develop a connection to events and historical accounts. This was aligned to the concept of historical empathy defined by Endacott and Brooks (2013) and Endacott (2014) and included *historical conceptualization, perspective taking* and *affective connections* as objectives. The reflective process and personal accounts of the Holocaust was a tool to support the development of historical empathy and the connection to professional identity (Kranz, 2014).

### Framework Approach and Qualitative Data Collection

The research project was approved by the University research ethics committee.

The research team adopted a ‘Grounded Theory’ framework (Marshall & Rossman, 1995; Strauss & Corbin, 1990), following Charmaz’s (2014) constructivist perspective, whereby the researcher is a ‘co-constructor of meaning’. Our aim was to avoid constraining the participants’ answers within predefined categories (Ashton & Bussu, 2020; Mills et al., 2006)

Qualitative data collection included three focus groups with students and semi-structured questionnaires with lecturers. The research team collected perceptions of personal experience in post visit focus groups.

Focus groups provide an opportunity for students to elaborate their personal ideas and to create connections between the past and the present (to support the development of historical empathy) and to express their personal views (Endacott & Brooks, 2013). Also, we consider these methods to be a form of peer education practice: all the participants were able to discuss the topic explored and shared personal opinions. The focus group method was thus adopted with students to support group discussion and to facilitate group reflection on the experience.

Lecturers’ perception of students’ personal experience (as well as pedagogic improvement strategies) was collected via semi -structured questionnaires.

#### Student Focus Group: Reflective Phase

In February 2017, 2018 and 2019 (post Holocaust sites visit), one focus group (2 hours) for each academic year, was conducted with students (*n* = 30). The focus groups explored participants’ perceived knowledge and awareness development (*historical contextualisation*), perceived development of factors consistent with the concept of historical empathy (*perspective taking* and *affective connections*) and explored ways to improve this experience in the future.

Each focus group was moderated by two experienced focus group researchers. A third researcher (observer) monitored group dynamics, the communication style of the facilitators, and the level of student engagement. The development and delivery of the focus groups supported and aligned to the criteria outlined by Krueger (2002) to ensure scientific rigor. The steps included:

- Inviting students by email to attend a meeting to explain the nature of the focus groups.
- Preparing a safe and comfortable environment at the University to conduct the focus group (including group boundaries and tolerance of other perspectives).
- Sharing focus group questions and roles within the research team (two moderators and one observer).

At the end of the focus group, the moderators and observer debriefed students and provided a summary of the most significant elements that emerged from the group (to check for clarification).

Importantly, the focus groups provided an opportunity for students to elaborate their personal ideas and to create connections between the past and the present (to support the development of historical empathy) and to express their personal views (Endacott & Brooks, 2013). This reflective process requires students to relate their “cognitive understanding” to their “affective connection” as Utami (2019) argues that teaching historical empathy can be achieved by such reflective learning modes. In this context, the role of the moderator was to facilitate exchange of opinions/views and encourage self-reflective thinking.

Each focus group was audio-recorded with names anonymised. The decision to audio-record the focus groups assisted the research process in two ways. Firstly, it allowed for a verbatim recording of the data to support rigorous computer-assisted qualitative data analysis. Secondly, it provided time for the research team to study the process and group dynamics.

#### Lecturer Semi-Structured Questionnaires

Semi-structured questionnaires (*n*=9), with open-ended responses to specific questions were distributed to the lecturers who visited Holocaust related sites with the students and/or delivered the pedagogic phase of the project.

### Qualitative Data Analysis

The focus groups (students) and semi-structured questionnaires (lecturers) were analysed with the software ATLAS.ti 7.5 (Friese, 2015), consistent with Grounded theory framework. That is, consistent with the Grounded theory framework, the researchers adopted an interpretative approach to reconstruct the representations and reflections of the participants (Bussu, 2016; Bussu et al., 2016; Charmaz, 2014; Niehaus et al., 2017). The use of the software ATLAS.ti assisted with data analysis by allowing for checking and rechecking of codes (conceptual development), then further cross-checking of findings from different codes (lecturers and students) with different methods (semi-structured questionnaires and focus groups).

**Fig. 1:**
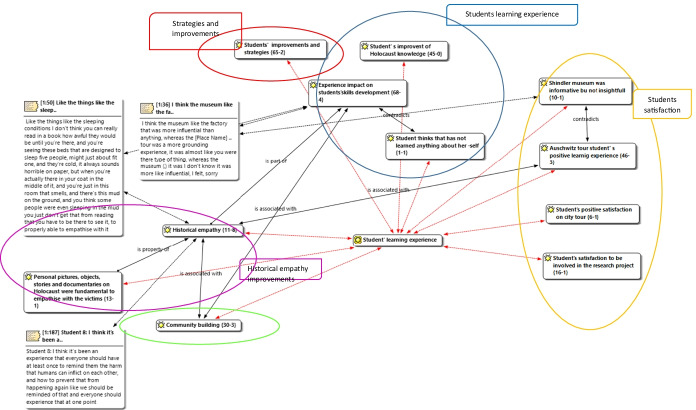
Students’s learning experience

As outlined in Figure 1, each box includes two numbers: the former represents the frequency of a given code within primary documents (PD) (focus groups and semi-structured questionnaires); the latter refers to the number of direct associations with other codes. Hermeneutic Unit (HU) provides the data structure for each project in ATLAS.ti and includes all data collected (focus groups and semi-structured questionnaires). ATLAS.ti network also assisted with the triangulation of data that emerged from focus groups and semi -structured questionnaires (Flick, 2004). To ensure a robust methodology, Seale’s (1999) research quality criteria was also respected (see Appendix 1) with theme saturation point (Sipman et al., 2019) reached after 3 focus groups and 9 semi-structured questionnaires.

## Results and Discussions

Once the analysis was completed, five key themes emerged. Factors related to Endacott (2014) conceptualization of historical empathy are inherent in the narrative*:* (1) Perception of learning development on Holocaust and contemporary issues (*historical contextualization*), (2) Perception of personal experience and empathic approach (*perspective taking* and *affective connections*), (3) Awareness of relationships with students and lecturers (*perspective taking*), (4) Factors impairing the educational setting (*perspective taking* and *affective connection*), (5) Improving the learning experience, and (6) Conceptual analysis: summary of codes and frequencies.

### Perception of Learning Development on Holocaust and Contemporary Issues (Historical Contextualisation)

The students’ valued the pedagogic sessions/phase positively as these were perceived as interactive sessions that provided a multi-disciplinary historical introduction of Nazism ideology and eugenics. Students also stressed that the tour (and visit) of Holocaust related sites in Poland, including the Auschwitz-Birkenau concentration camp, assisted in solidifying and contextualising prior knowledge developed as part of the degree (Q1 and Q2, Table 1). Pier (a lecturer) (Q2) more directly linked the Holocaust visit to the broader university degree.

**Table 1:** Perception of learning development on Holocaust and contemporary issues

Focus Group Quotations	**Q1** Andrea: I think it was the setting stone for a lot of interactions from the United Nation and that sort of thing but I still don’t think it’s any better today that it was seventy years ago, eighty years ago…I still don’t think there’s enough globalised law to stop it from happening, so I can see why it happened but obviously when you’re there in person, it’s like you sort of question how the hell did this get so big, so quick and not get stopped for two, three years. I still think it could happen today and it would take too long and too many people to die before it was stopped.
	**Q2** Pier: Students have a session about the Nuremburg trials and the development of the International Criminal Court, so it’s good for them to have some more background to these events. The 1st years also have sessions on victimology and human trafficking in 2nd year, so these topics can further link in with what students learn on this trip.
	**Q3** Giselle: I think it’s made my experience in university a lot better as well looking at things differently, like [Person Name] … said before about a Nazi’s point of view about family and stuff like that, like I wouldn’t have thought about that, but [Person Name] … thought of it, so it’s just I really enjoyed it.
	**Q4** Nina: I think until you go there and experience it like walking round and that you don’t realize how much of an impact it will have had on like society today, you know like the changes that we’ve made since (…).
	**Q5** Pablo: I think that introducing the students to the Holocaust is very beneficial because it helps to develop empathy and understanding of other group (…) This makes the Holocaust an important part of our history, which means it should be taught.
	**Q6** Maria: You could read it all yourself. She was not really telling you much anyway. I could not understand her anyway. (…) We were a such a such a big group as well the tour guide was talking about something and half of us had not even got to that point yet.
	**Q7** Amanda: I just think it makes you look at today’s world, and it makes you think that we need to put more things in place to stop it ever happening again, like obviously we’ve got dictators in power like [Person Name] …, and just everyone it makes you just take a step back of the world and appreciate what you’ve got and just think you actually need to take a step in doing something to counteract just anything basically, like even if it’s means just something as simple as actually voting in the referendum it just makes you think that every little voice does matter (…).

Central to contextualising knowledge and considering diverse views, was an understanding of how these events connect to students today (Q3 and Q4 Table 1).

This connectivity to the past via Holocaust visits was supported by research by Starratt et al. (2017) and Endacott and Brooks (2013) who demonstrated evidence for the impact of Holocaust visits for the development of citizenship and anti-fascist movements by fostering connection between historical events and the current socio-political context. Indeed, the connection between the past and present is also an important learning aspect of the pedagogy of remembrance and involves a combination of cognitive, affective, and pragmatic elements (Kranz, 2014; Nieuwenhuyse & Wils, 2012) as a precursor to the development of historical empathy (Endacott, 2014). This affective connection to personal narratives, according to Eisenberg (2000) is however linked to our competency to understand other perspectives and varying personal attitudes. Feedback from lecturers’ and students’ indicates understanding and supportive personal attitudes are necessary for the development of historical empathy. This was further supported by a lecturer (Q5, Table 1).

Thus, students and lecturers highlighted the importance of remembering and commemorating the Holocaust to promote tolerance, empathy and understanding of others as pre-cursers to new forms of genocide (Delasalas, 2014).

According to Hoon (2014) the question of empathy in teaching and learning about Holocaust is an important issue that needs to be raised and explore more, especially the impact that visiting Holocaust related sites students can have on both students and lecturers.

The students’ emotional connection to events at Auschwitz-Birkenau was enhanced by reading the stories of the detainees, seeing their personal possessions, visiting where and how they lived, and activities they conducted daily. Students’, however, perceived the visit to the Schindler’s Factory Museum as less emotionally engaging and less informative than the Auschwitz-Birkenau camp visit.

This is likely to be due to factors related to “museum spaces and tour management”. That is, the museum was overcrowded, noisy, and the guide was difficult to hear. Given these factors, students felt that they had did not have enough time to read and comprehend the collections and documents exhibited in the museum. This is also reflected by Maria below (Q6, Table 1). This is relevant aspect that needs to take in consideration to museum curators for efficiently managing visitors flows, considering the impact on students learning experience.

Although the experience facilitated understanding of diverse perspectives of the Holocaust, many expressed their difficulties in understanding this “terrible tragedy” with some students openly struggling with the notion that this was “allowed to happen”. Despite difficulties comprehending such events, the students overwhelming understood the importance of visiting such sites and preventing new movements of nationalistic eugenics and genocide (Levene, 2000) (Q7, Table 1).

### Perception of Personal Experience and Empathic Approach (Historical Contextualisation, Perspective Taking, and Affective Connection)

Consistent with research by Erber (2002), students were able to reflect on the impact of state manipulation and social conformity as a precursor to genocide (Q8, Table 2).

**Table 2:** Perception of personal experience and empathic approach

Focus Group Quotations	**Q8** Giselle: When I think about it I always think like in that day if I’d had been brought up in Poland, and I was brought up to like think that Jews were bad people, and thought that was going on would I have just thought oh well you know they’re Jews, they’re bad people, I’d like to think I wouldn’t, but when you’re living in that sort of environment, and that sort of society like you don’t know what you’d do, but I learnt that. It’s that people are out there, and I think that could happen again even in this day and age…
	**Q9** Erin: I think it was shocking like the scale of how it actually like I do not think any of us were prepared …is just unthinkable” (…) It was very informative, (.) and you did like appreciate at a meal when we all left the food and that, and when we’d seen and looked at the pictures, and how they gave them a piece of bread and they told them they couldn’t finish it all in the morning because they had to come back and eat exactly the same, but like we left plates and plates of food (h) you just appreciate what you’ve got.
	**Q10** Catherine: Kid ‘shoes and stuff, you can’t comprehend, can you? Like you do not have a kids but obviously you will have kids in your family or whatever and you just see them as innocents and there were someone’s shoes (…) It was the shoes that got me because there was like a pair of little girls shoes right at the front, it was just like could not be more than 3 years old (…) How could you cause so much pain when someone ‘so small? (…).
	**Q11** Freddy: I still can’t look back at the pictures that I’ve took, it’s just almost traumatizing in a way, I don’t want to look back at the pictures like force myself to think about it, I’ve just sort of since I’ve got back I’ve just sort of trying to forget about it because I don’t want to force myself to actually consider it all again?.
	**Q12** Giselle: I think definitely the listening and definitely the empathy because I feel when we were all walking round no one was talking, everyone was just so focused just completely listening and empathy I think that’s just obvious like it forced everyone to just stand and think about what it was like.
	**Q13** Matilda: When you’re actually there in your coat in the middle of it, and you’re just in this room that smells, and there’s this mud on the ground, and you think some people were even sleeping in the mud you just don’t get that from reading that you have to be there to see it, to properly able to empathize with it.
	**Q14** Erman: I think this experience can develop understanding and empathy, concern for other people. I think it can also help students to think about how to relate to and communicate with different groups, marginal groups and their role in ending discrimination and unfairness.
	**Q15** Erman: This experience it was very important for me and my profession. I have reflected more about factors link to empathy. I observed myself to understand which strategies I use to create a co-constructive relation with students and colleagues when there are “language barriers” and “culture barriers”.

One student highlighted how this experience was shocking, but, at the same time, relevant and insightful today (Q9, Table 2). That is, by visiting Holocaust related sites students were able to connect with the victims’ personal accounts and make relevant to themselves. These are perceived as important precursors to historical empathy (Endacott & Brooks, 2013)

To expand on this, students reflected on human rights abuse and related this to themselves by questioning ‘how it was possible committed impunity crime against innocent’ victims’ and without empathy for them. For the students, the integration of curriculum and experiences helped to connect historical context of the Holocaust with their present lives and experiences, rather than compartmentalizing the Holocaust experience as something distal and external from themselves.

This stance is consistent with studies by Cowan and Maitles (2011) who demonstrated the link between the impact of pedagogic content about Holocaust sites to contemporary genocides and human rights. Indeed, this connection to contemporary issues was facilitated by personalisation of the victims via mediums such as photographs and personal daily items (e.g. shoes, hair) (see for example the quotation 10, Table 2).

Students were able to identify with the suffering of the detainees and how they prepared themselves for death. Adjectives used by the students to describe recurrent images related to the Holocaust were highly emotive and powerful (Q11, Table 2).

This also highlights the importance of reflection and individualised coping strategies to support students in developing personal skills such as tolerance and empathy. For example, Cowan and Maitles (2011) described the need for students to immerse themselves in the “real experience” (learning by doing) to enhance their personal development. By immersing themselves in the experience, students’were able to express the emotional and cognitive impact the experience was having on them (Q12, Table 2).

Students highlighted that they had become more aware and developed enhanced critical thinking skills (a questioning approach) on the Holocaust issue and on social discrimination broadly. One critical aspect highlighted by the participants was the development of a questioning approach (critical analysis), increased tolerance of other views, and affective connection to the victims of the Holocaust (historical empathy) (Cowan & Maitles, 2011; Delasalas, 2014). That is, affective connection and tolerance (fostered via sensory experiences) was important as many would work in the social justice system (Barton & Levstik, 2004; Botvin & Griffin, 2004) (Q13, Table 2).

Similarly, the perceptions of the lecturers supported the Holocaust visit as important for students to develop empathy, tolerance and to highlight broader issues such as advocacy and social consciousness (Q14, Table 2). Lecturers also highlighted how the reaction of students to the visit had a profound impact on their own experience and how the experience helped them personally to reflect and think empathetically about the consequences of the Holocaust (Q15, Table 2).

### Awareness of Relationships Between Students and Lecturers (Perspective Taking)

From the student’s perspective, sharing personal emotions and opinion with other students, and students and lecturers, during the journey, has contributed effectively to students learning experience (see students Q16 and Q17, Table 3) (Romi & Lev, 2007).

**Table 3:** Awareness of relationships with students and lecturers

Focus Group Quotations	**Q16** Nina: It made everyone closer I think like it needs to happen sooner in the year as opposed to third year. we shared such an emotional experience.
	**Q17** Lilly: Brought us closer, I don’t think I’ve ever spoke to you, but then people who you don’t really like speak to, like do things together because you’re all like …I think we socialised with a lot more different people that we wouldn’t necessarily socialise with on our course on that trip which was nice.
	**Q18** Amanda: Well they didn’t have a role, we kind of took away like their lecturers and just kind of like bonded with us as just people, it didn’t feel like we were talking to our lectures, I just felt like we were talking, just having a conversation.
	**Q19** Julianne: Yeah, I think the whole experience like how we were saying we’ve grown closer as a group because of the experience I think we grew closer to the tutors that came with us.
	**Q20** Lilly: I do not think I learnt any think new about myself (..) I think we have socialised with a lot more different people that we would not necessarily socialise with our course on that trip which was nice.

Furthermore, for the students, sharing this experience outside the formal academic environment with their lecturers and other students consolidated their interpersonal relationships. This is supported by research which indicates that interpersonal relationships between (& among) students and lecturers fostered outside the formal education environment can influence the learning experience in a positive way by creating spontaneous learning opportunities and emotional connections to the content (Karn, 2012; Nowell & Poindexter, 2018).

Students noted how it was insightful to see their lecturers in a different (more personal) and often spontaneous role outside the university setting. Thus, by participating in this shared activity, the students developed a bond with students and lecturers who some had previously not engaged with. Students and tutors bonded in way that was less formal and more relaxed allowing for enhanced group collaboration and group reflection, supporting tolerance and understanding of varied perspectives (Q16 and Q17, Table 3).

Research indicates that sharing personal reflections and emotions related to the Holocaust in informal groups, outside the classroom, is important for fostering student connection to historical events (Nieuwenhuyse & Wils, 2012). This was supported by Banks (2008) who found that it was easier for participants to create and foster a “group identity” via an emotional shared external visit, than after three years of university-based teaching. Thus, a practical recommendation is that such learning experiences should be planned, where possible, for first year undergraduate students, in order to foster and promote community building, and that participants be able to share opinions and emotions in informal situations before and after the visit to Auschwitz-Birkenau. During this visit, students and lecturers shared social events such as lunch and dinners on the trip to enhance group cohesion and the learning process (Perez et al., 2012) as outlined below (Q20, Table 3). It could however, be that group learning and cohesion was more directly related to the formative pedagogic phase (university based or informal aspects outside site visits) rather than the accompanying Holocaust site visits.

Holocaust Education should however, not be a place where teachers force their values and world views onto students. Holocaust education should ‘stress the formulations of values’ by students and this should emerge from their own through critical thinking and reflection processes (Karn, 2012, 235).

In summary, “group identity” was fostered and sustained via a process of reflection and sharing emotional experience (beyond any other university experience), with student focus groups also enhancing the “group experience” by providing a safe environment for students to share their perceptions, opinions and emotions.

### Factors Impairing the Educational Setting (Perspective Taking and Affective Connection)

Some lecturers and students aligned the Holocaust visits to scheduled and detached tourist attractions, particularly in Krakow. At Auschwitz-Birkenau, students were not able to explore the site individually (away from guides) to reflect on the significance and affective perception of the visit. Consistent with this view, some students felt that people were treating Auschwitz as a ‘tourist attraction’ rather than a sensitive memorial site (Q21, Table 4).

**Table 4:** Factors impairing the educational setting

Focus Group Quotations	**Q21** Andrea: I think those people saw it more as a tourist attraction though because they were the people that were taking pictures in front of things whereas we weren’t doing that. So, I think that was more the people who saw it as a tourist attraction.
	**Q22** Sonia: I didn’t expect the tour guides to be so matter of fact about things like this happens, that happened, I expected them to be more emotional, but I suppose they’re just used to it you know like because they’re taking people on tours every day, and I expected them to be more emotional, because it was like their people who died.
	**Q23** Andrea: I think those people saw it more as a tourist attraction though because they were the people that were taking pictures in front of things whereas we weren’t doing that. So, I think that was more the people who saw it as a tourist attraction.
	**Q24** Freddy: I think for me the only thing I would change is the Tour. I feel like we might have enjoyed it more and got more out of it if we was allowed to go around by ourselves you know instead of having the tour guide … and there was no time to like stop and take pictures.
	**Q25** Sara: I was by myself for a bit. I feel like that was better. I said when we came back that was my time to take it in. It was when we were walking from the entrance down to all the memorial bits down at the bottom next to all the track and I just wandered off because I decided to walk by myself and that was better, I got a bit overwhelmed then because I was a bit like, when you’re by yourself, not having everyone chatting away it is a bit like oh god. You realise where you are and what’s going on and stuff(…).
	**Q26** Franckie: However, the silence on the journey home from Auschwitz gave me the impression that the students were sitting quietly, reflecting on what they had experienced, which in that respect suggested that students had been touched by visiting the concentration camp.
	**Q27** Andrea: I think for me it was quite overwhelming immediately afterwards because obviously not only have you just experienced all that history, you’re also shattered because you’ve just walked round all day so obviously your guard’s down and you’re just like oh my god this has just been overwhelming but the now that I’m reflecting on it a couple of weeks later, I think I still benefited from it(…), being let off by ourselves I think we would have reflected on it a lot more(…).

The link between tourism and the Holocaust was made by researchers who developed the term “dark tourism” to include sites associated with death, suffering and the macabre (Griffiths, 2019; Stone, 2012). “Dark tourism” has been linked to negative factors that hinder learning and development (Stone, 2012) by impeding personal awareness (Davis & Rubinstein-Avila, 2013). For example, Griffiths (2019) found that “dark tourism” “commercialization” an “emotional disconnection” (Griffiths, 2019) to such sites might paradoxically reinforce existing prejudices rather than providing a transformative learning experience (Bastel et al., 2015).

Consistent with this theme, the guided tour was considered as “fast” and "cold" and “impersonal” by some students. This aspect was perceived as asymmetric with the reflective and emotional needs of the students during the visit, for example Q23 and 24 (Table 4).

The students also added that younger visitors (primary and secondary school students) were perhaps too young and hence not cognitively and emotionally prepared to fully understand the significance of visits to Holocaust sites. This highlights the need to actively involve young visitors in pre-visit (& during the visit) pedagogic content to prevent a passive reflective stance (Cowan & Maitles, 2011).

Students would have appreciated more time “for themselves” (during and after the visit) to reflect on their personal experience to enhance knowledge and learning through awareness (Davis & Rubinstein-Avila, 2013). This thematic concept was repeated several times by students, who acknowledged the importance to be able to have “personal space” to understand the experience. Freddy outlined the implications of personal time and space during the visit (Q24, Table 4).

Sara, Andrea and Franckie advocated “personal time and space” after the visit (immediately and ongoing) to support their learning experience (Q25, Q26 and Q27, Table 4).

### Improving the Learning Experience (Historical Contextualisation and Perspective Taking)

According to Cowan and Maitles (2011), indoor and outdoor learning experiences can improve awareness on Holocaust and genocides, and hence empower students to become responsible citizens. It is therefore important that Holocaust educational experiences consider students’ needs and identify best practice for future visits (Q28, Table 5).

**Table 5:** Improving the learning experience

Focus Group Quotations	**Q28** Tina: (…) It is very important to collect training needs of students. Sharing together a handbook of best practices and to promote a satisfactory learning experience.
	**Q29** Freddy: I think for me the only thing that I would change is you know the [Tour Name]. I feel like we might have enjoyed it more and got more out of it if we was allowed to go around by ourselves you know instead of having the tour guide, we were really moving quite quickly and there was no time to like stop and take pictures, and actually read the things, because the lady was just sort of you felt a bit rushed around.
	**Q30** Elvis: I think that this is a brilliant trip. The guide on the Auschwitz and Birkenau tour said that Auschwitz survivors occasionally give talks to visitors, but I presume this is not t very often and I do not know how much it would cost, but it might be worth considering if possible.
	**Q31** Maria: It felt for me like at that time and like when we were there it felt surreal, like it didn’t happen, but then it was after like when I got back to the hotel that it hit me and I was like, I just felt like depressed, and I was like just going through my head everything what we heard.

As previously discussed in 3.4, students highlighted the importance of time with specific reference to the tour and the tour guides (Q29, Table 5). Students discussed how the experience could be improved by having more time with the tour guides and having time to “absorb” the emotional experience on the tour to make relevant to the broader issues in contemporary society.

A future recommendation of the project is to incorporate timetabled reflective space and time to support student development during and after the visit (Delasalas, 2014). This could include written reflective accounts (e.g. on-line portfolio) aligned to professional values and identity of a student.

Lecturers provided further recommendations to enhance the pedagogical experience for future cohorts of students. This included a brief overview of the trip on the morning of the visit to students about “what to expect” and reference to expanding the pedagogic content prior to the visit (by offering tips and advice about the trip; and by viewing footage of former inmates of Auschwitz) (Q30, Table 5).

The research highlighted that this experience cannot be implemented without reference to the context of the course, with key concepts and experiences that emerged from the Holocaust visit meaningfully revisited throughout the duration of the course (such as linking to recent genocides and human rights abuse). This would facilitate a connection between the student’s identity (personal and professional), and the degree curriculum (in this case integration of the disciplines of psychology with applied sociology of deviance). This connection was facilitated by the degree curriculum and placement experiences during the course to include communication skills in the context of offenders and victims of crime and placements with vulnerable groups in the sector (e.g., young offenders). Therefore, given that the course is embedded in personal narratives and connections with vulnerable people, it is likely that development of historical empathy was facilitated prior to this visit. Despite this direct and explicit links between student’s professional identity and the degree, some students (3^rd^ year focus group) could not identify a clear connection between their future professional identity and their learning related to the Holocaust.

Given the sensitive and complex nature of issues explored as part of the degree, emotional and interpersonal skill development is inherent in the program (Botvin & Griffin, 2004).

The findings also suggest that lecturers pay attention to their own behaviour in order to support students in their emotional elaboration (Schunk, 2005). Lindquist (2011) argues that the lecturers’ behaviour in confronting an emotive topic can heavily influence student reactions. In consideration of this aspect, lecturers need to be adequately prepared and remain adaptable and professional, by not being overcome by their own emotional responses. The potential to cause unmanageable distress is outlined in quotation Q31 (Table 5).

In summary, future visits should incorporate opportunities for participants to share their emotional experiences with the rest of the group with lecturers who are specialist in Holocaust education (Hen & Sharabi-Nov, 2014). Consistent with the concept of emotional pedagogy (Zembylas, 2013) experiences and themes (such as human rights) must be meaningful and revisited throughout the duration of the degree programme to allow for emotional connectivity and currency.

### Conceptual Analysis: Summary of Codes and Frequencies

The last section presents the most frequent, relevant and inter-connected narratives related to student learning experience from students ’ point of view, analysed in the previous sections. The network (Figure 1) synthesizes the most important dimensions emerging from the qualitative data analysis

1. *Student satisfaction. (historical contextualization, perspective taking and affective connections).* Most students were satisfied with the experience. That is, students have recognised the value of “Auschwitz tour student’s positive learning experience” (code, 46 quotations Fig. 1), with most students outlining that this was more emotionally engaging. Furthermore, students have enjoyed the city tour (code “Student’s positive satisfaction on city tour”, 6 quotations) and they recognised benefits of being involved in the research to enhance understanding of the research process (code “*Student’s satisfaction to be involved in the research project code*”, 16 quotes, Fig. 1). In comparison with the Auschwitz-Birkenau visit, “Schindler Factory Museum was considered less insightful but informative”. Students perceived less personal connection and hence less emotional responses/impact to the “Schindler Factory Museum” due to factors related to “museum space and management” (see 3.2 code, 10 quotations, Fig. 1, and “Perception of Learning Development on Holocaust and Contemporary Issues (Historical Contextualisation)”, and “Factors Impairing the Educational Setting (Perspective Taking and Affective Connection)” sections).
2. *Student learning experience (historical contextualization, perspective taking and affective connections):* Students have improved their knowledge about the Holocaust and associated genocides (code “*Student’s improvement* on Holocaust knowledge” is associated with 45 quotations (Fig. 1) Furthermore students have perceived an improvement in their interpersonal skills (code “Experience impact on student’s skills development, 68 quotations) with only one student indicating that the activities did not have any impact on his/her skill development) (Fig. 1, “Perception of Learning Development on Holocaust and Contemporary Issues (Historical Contextualisation)”, “Perception of Personal Experience and Empathic Approach (Historical Contextualisation, Perspective Taking, and Affective Connection)” and “Factors Impairing the Educational Setting (Perspective Taking and Affective Connection)” sections).
3. *Historical empathy (historical contextualisation, perspective taking and affective connections):* Although all concepts relate to historical empathy conceptualization, explicit relevance is highlighted within this section with direct quotes (*n* = 11) and also related to a number of other codes (*n* = 10). This highlights the complexity of the concept and its link to emotional and social development. The *historical empathy* was facilitated via a process of personalising the experience by exposing students to personal pictures, personal objects, and personal stories & documentaries (Personal pictures, objects, stories and documentaries on Holocaust were fundamental to empathized with the victims code is associated with 13 quotations) (Fig. 1, “Perception of Personal Experience and Empathic Approach (Historical Contextualisation, Perspective Taking, and Affective Connection))” section.
4. *Community building (perspective taking) and affective connections):* Another important concept included interpersonal relationships and community building (code associated with 30 quotations) (Fig. 1, “Awareness of Relationships Between Students and Lecturers (Perspective Taking)” section).
5. *Improvements (historical contextualisation, perspective taking and affective connections)*: Finally, as previously discussed, students (65 quotations from *student’s improvements and strategies*) discussed future recommendations to improve their learning experience. This included time and space for reflection and the associated opportunity to revisit core concepts longitudinally throughout the course (“Improving the Learning Experience (Historical Contextualisation and Perspective Taking)” section).

### Conclusion and Implications

The study is innovative, as few qualitative studies have explored undergraduate students perceived learning experience visiting Holocaust -related sites. The current literature is predominately focused on the pedagogical impact of Holocaust education, and/or visiting Holocaust-related sites by primary and school children. There is a lack of evidence the impact of (or perceived impact) of visiting Holocaust-related sites in higher education. Those studies within Higher Education have tended to focus on students training as medics or nurses (Adams et al., 2006; Ben-Sefer, 2006; González-López & Ríos-Cortés, 2016; Reis et al., 2019) or have adopted quantitative approaches (Romi & Lev, 2007).

The research design actively encouraged and supported students in reflecting on this emotional experience. That is, the research process itself fostered engagement in the learning process by providing a forum (particularly focus groups) to elaborate on knowledge and reflect on experience. The research also provided an opportunity for students to be practically involved in research on pedagogic processes, which is central to the degree course and their professional development.

While the research found that the course related Holocaust visit (and pedagogic learning) has improved student perceptions of their learning experience and understanding of the Holocaust, it is not clear if this impact is sustained long term.

Although there are limitations to the study and the Holocaust educational programme (such as lack of reflective time and space), this pedagogical programme could easily be replicable with other cohorts of university students provided that the experience is embedded in course content and experiences (e.g. placements) and extended to staff working at Holocaust related sites. That is, expanding this educational programme to other disciplines also requires that educators link the curriculum to personal narratives throughout the course, as a means to facilitate the development of historical empathy.

Learning about and reflecting on the importance of moral and ethical principals’ during Holocaust visits (& curriculum) poses valuable questions and issues for the education of health professionals. What is particularly important is that health professionals reflect on racism, ethics, and how/why health institutions/societies supported mass genocide (Reis et al., 2019).

In recent years this has led to inter-professional higher education courses that specifically focus on the Holocaust and the development of key policies and declarations. The Galilee Declaration and the Stockholm Declaration called for curriculum that explicitly focuses on the role of healthcare professionals in the Holocaust, and that such curriculum should be compulsory for all healthcare training programs (Declaration of Stockholm, 2000; Reis et al., 2019). The authors of this paper suggest that this declaration should be extended to related health and social care programmes in Higher Education.

It is recommended that future research is required to examine the impact of the Holocaust experience longitudinally on the development of student’s historical empathy and professional identity. Furthermore, as personal identity cannot be separated from the development of historical empathy in this context, further research and educational programmes should explore how students and lecturers’ “personal identity self- identification” (i.e German, Jewish, LGBTQ) impacts on experiential learning and engagement (Lazar et al., 2004a, b; Mayes, 2010; Nager et al., 213).

Additionally, considering covid -19 pandemic, it is recommended that future research will explore the impact of Auschwitz Memorial virtual tour (recently available) on students learning processes and compare this effectiveness with the traditional tour. There is little doubt that during the pandemic, technology has improved the quality and impact of virtual tours. Despite this, evidence suggests that classroom lectures are significantly better than virtual tours/reality in terms of knowledge acquisition, but that virtual reality/tours are more important in developing empathic responses to lived experience (Richards et al., 2021).

In support of this, findings from this research indicate that students cannot be passive in any process (virtual or face-to-face tour) that promotes and facilitates historical empathy. In the context of virtual tours, students should still be supported by pedagogic content from a variety of sources (such as traditional lectures), include account and narratives of ordinary people, and manage conflicting sources in order to recognise “authentic” application of knowledge (Sweeney et al., 2018). Furthermore, any virtual tour should include the opportunity and “space” for students to immerse themselves with the Holocaust, and include structured reflections (such as focus groups, diaries) that provide the opportunity to revisit and ask questions. Lecturers should be educationally qualified and experienced in Holocaust education, be willing to challenge images and accounts proposed by students and be emotionally attune to students. Only via such meaningful affective engagement will students develop historical empathy.

Further research is therefore not only required on the impact of virtual tours/reality on students learning process at Holocaust sites during the pandemic, but the impact of virtual tours in combination with traditional pedagogic delivery/content also requires exploration.

## Appendix 1

**Table:**

*Seale’s qualitative criteria (1999):*
1. ***Credibility*** (internal validity), member validation or validating findings where the participants assess how much they can relate to the researcher’s construct of the phenomenon.
2. ***Transferability*** (external validity): A description of the method was provided along with detailed information on all the research process.
3. ***Dependability***: All the research stages and methods were documented in order to allow an assessment on the propriety of the whole procedure. A description of the methods was provided.
4. ***Authenticity***: The participants, students and lecturers, could develop greater understanding of the phenomenon and through the focus group and semi -structured questionnaires could compare different perspectives. The research also encouraged improvements suggested by participants.
5. ***Confirmability***: The research team shared the methodology, coded and interpreted the information (internal confirmability). The research is replicable (external confirmability): The interpretation of data was shared by the team.
